# Recent Updates of DNA Incorporated in Carbon Nanotubes and Nanoparticles for Electrochemical Sensors and Biosensors

**DOI:** 10.3390/s8117191

**Published:** 2008-11-13

**Authors:** Umasankar Yogeswaran, Soundappan Thiagarajan, Shen-Ming Chen

**Affiliations:** Department of Chemical Engineering and Biotechnology, National Taipei University of Technology, No.1, Section 3, Chung-Hsiao East Road, Taipei 106, Taiwan (ROC);

**Keywords:** DNA, carbon nanotubes, nanoparticles, electrochemical sensors, biosensors, hybrid

## Abstract

Innovations in the field of electrochemical sensors and biosensors are of much importance nowadays. These devices are designed with probes and micro electrodes. The miniaturized designs of these sensors allow analyses of materials without damaging the samples. Some of these sensors are also useful for real time analysis within the host system, so these sensors are considered to be more advantageous than other types of sensors. The active sensing materials used in these types of sensors can be any material that acts as a catalyst for the oxidation or reduction of particular analyte or set of analytes. Among various kinds of sensing materials, deoxyribonucleic acid (DNA), carbon nanotubes (CNTs) and nanoparticles have received considerable attraction in recent years. DNA is one of the classes of natural polymers, which can interact with CNTs and nanoparticles to form new types of composite materials. These composite materials have also been used as sensing materials for sensor applications. They have advantages in characteristics such as extraordinary low weight and multifunctional properties. In this article, advantages of DNA incorporated in CNT and nanoparticle hybrids for electrochemical sensors and biosensors are presented in detail, along with some key results noted from the literature.

## 1. Introduction

Sensors can be broadly classified in two categories: chemical sensors and biosensors. The biosensors can be defined in terms of sensing aspects, where these sensors can sense biochemical compounds such as proteins, nucleotides and even tissues of living organisms [1-3]. Similarly, the chemical sensors are used for the detection of various chemical compounds. Within these sensors, the active sensing material on the electrode should act as a catalyst and catalyze the reaction of the compounds to obtain the output signals [4, 5]. Generally, the working principle of these two sensors is based on the electrochemical method used in the process [6-8]. The selection and development of an active sensing material is one of the challenges here. The active sensing material should act as a catalyst for the oxidation or reduction of particular analyte or set of analytes. The recent developments in the nanotechnology have paved the way for large number of new materials and devices of desirable properties which have useful functions in numerous electrochemical sensor and biosensor applications [9-13]. Basically, by creating nanostructures it is possible to control the fundamental properties of materials even without changing the chemical composition of the later. In this way, an attractive world of low dimensional systems, together with the current tendencies on the fabrication of functional nanostructured arrays has risen in the field of nanotechnology. Further, the nanostructures can be well used in the processes of efficient transport of electrons and in optical excitations and these two factors make them critical to the function and integration of nanoscale devices [14-16]. In fact, nanosystems are the smallest dimensional structures which can be used for efficient transport of electrons and thus they become essential to the function and integration of the nanoscale devices. Due to their high surface area which is associated with nanometer-sized materials, a tremendous improvement in electrochemical properties is achievable along with the reduction in size of these devices [17]. Generally, these kind of nanostructures could be prepared by two methods, one is by “bottom-up” approach where, the self-assembly of small sized structures form into larger structures. The other is by “top-down” approach where, the reduction of large systems down into smaller sizes to produce multifunctional nanoscale structures [18, 19].

Recent studies have shown considerable attraction towards these nanostructures, particularly on carbon nanotubes (CNTs). CNTs are rolled up graphite sheets that are in nano scale tube form. After the discovery of carbon nanotubes by Iijima [20], various types of CNTs such as single-walled (SWCNTs), double-walled (DWCNTs) or multi-walled (MWCNTs) graphene tubes have been produced by various methods like chemical vapor deposition (CVD) and arc-discharge [21]. The properties of CNTs allow them to interact with some organic aromatic compounds through π-π electronic and hydrophobic interactions [22-25]. These interactions are used for preparing composite sandwiched films for electrocatalytic studies [26-29] and in the designing of nano devices with the help of non-covalent adsorption of enzyme and proteins on the side walls of CNTs. This has resulted in novel CNTs based nanostructures which contain chemical and biochemical units in them [30, 31]. For example, in our various work, the functionalized MWCNTs have been used for the preparation of composite films. The active materials used with functionalized MWCNTs are poly(neural red), Nafion, metal nanoparticles, cytochrome *c* (cyt *c*) and hydroxypropyl-β-cyclodextrin. The MWCNTs-poly (neural red) has been used for NADH, neurotransmitters, (-)-epigallocatechin gallate, halogen and sulfur oxyanion sensors [10, 13]. Similarly, MWCNT with cyt *c* composite has been used for the detection of halogen oxyanions, ascorbic acid and L-cysteine [16]. The other active materials with MWCNT mentioned above have been used for the simultaneous determination of neurotransmitters with ascorbic and uric acid [9, 11, 15].

Besides CNTs, nanoparticles possess unique physical and chemical properties. Previous studies unleashed various possible applications of such nanoparticles in the development of information storage, photovoltaic and biosensor devices [32]. Such kinds of nanometer size materials are gold [33, 34], SiO_2_ nanoparticles and the like [35, 36]. For example, Ikeda *et al.* demonstrated that gold nanoparticles could self-assemble both inside the network and on the surface of the silica gel [37]. Willner *et al.* explored the possible use of Au array for the construction of an electrochemical sensor with an extraordinary electrocatalytic property towards the electrochemical reduction of molecular oxygen in acidic media [38].

Further, the literature survey has shown that the combination of nanomaterials and biomolecules is of great interest in the field of electroanalysis. Nanoparticles could play an important role in immobilization of biomolecules due to their large specific surface area, excellent biocompatibility and good conductivity. The important components of such nanoscale systems composed of biomaterials are proteins or enzymes, antigens or antibodies, receptors and deoxyribonucleic acid (DNA). These nanoscale systems demonstrate unique catalytic or recognition properties [39]. The immobilization of these proteins on nanostructured materials such as CNTs with colloidal gold hybrids, montmorillonite, clay, mesoporous materials and molecular sieves have been identified as very promising methods for biosensing applications [40-45]. In the literature, electrochemical sensor device designing, selection of suitable sensing material for suitable analyte and their wide applications are already abundant [46-49]. Among these, application of CNTs in biosensors has been seen as the most interesting study [50-54]. However, detailed literature survey reveals that until now no one has attempted to present a review on DNA with CNT and nanoparticle hybrids as an active material for biosensing applications. In this review, we are especially interested in reporting about the electrochemical sensor and biosensor devices made of DNA incorporated with CNT and nanoparticle hybrid films as active sensing materials.

## 2. Purification and Functionalization of CNTs

Various creative methods have been employed in the preparation of CNT modified electrodes. Among them, in the preparation, the process involves one step or multi-step procedures. The CVD growth of CNTs on the carbon paper and cutting this paper to a suitable size and then placing it on a teflon jacket with an electrical contact provides a simple direct preparation of CNT modified electrodes [55]. Soft lithography mediated CVD and plasma assisted CVD have also been used to prepare CNTs on Si or PDMS substrates and were applied directly as working electrodes [56]. A flexible and aligned CNT electrode has been prepared by Chen. *et al.* where, numerous steps have been involved such as spin coating of iron (III) tosylate on CNTs which is followed by chemical vapor phase polymerization of 3,4-ethylenedioxythiophene and casting of poly (vinylidene fluoride), and then finally pealing of the electrode from the quartz supporting plate [57]. Other than the above mentioned electrode preparation methods, CNT modified disk electrode preparation methods [58], Ni nanoparticles assisted plasma enhanced CVD methods [59] have also been reported in the literature.

So far, the direct preparation methods of CNT modified electrodes from the CVD chamber have been explained. In the later decades, scientists reported that the quantities of carbon and metallic impurities in the raw materials are often too high, requiring post synthesis purification steps which should be used to remove them. The effect of metallic impurities present in raw CNTs can be listed as deagglomeration of bundles and ropes, CNT dispersion, alignment and interfacial bonding. Numerous reports have been made on the post synthesis purification of CNTs. It is obvious that the effective utilization of CNTs in composite applications depends strongly on the ability to disperse the CNTs individually and uniformly throughout the matrix without destroying their integrity or reducing their aspect ratio. The preprocessing of CNTs after the synthesis could be explained by the following three steps; purification to eliminate non-nanotube material, deagglomeration for dispersing individual nanotubes and chemical functionalization for improving nanotube/matrix interactions for processability with property enhancement. Briefly, in the purification process, the difference in oxidation rate between carbon nanotubes and carbon by products is an advantage, where the oxidative treatments are helpful in removing the impurities. These purification procedures are further classified in to wet oxidation and gas phase oxidation. The wet oxidation refers to the oxidation using a solution containing strong oxidizing reagents such as strong acid or H_2_O_2_ [60-63]. The gas phase oxidation is done using air or oxygen [64-66]. A typical example of wet oxidation would be as follows: CNTs (about 100 mg) are dispersed in 6.0 M HCl (60 mL) for 4 h under ultrasonic agitation, then washed until the pH of the solution is neutral and finally dried. In the same article, another purification method was also proposed follows: the CNTs have been dispersed in 2.2 M HNO_3_ (60 mL) for 20 h at room temperature with the aid of ultrasonic agitation (for 30 min), then washed with distilled water to neutrality and dried in an oven at 37 °C [67]. The CNT purification has also been accomplished by stirring the CNT in concentrated nitric acid at 25 °C for 24 h [68]. Other than the above wet oxidation methods, numerous acid treatments are also available in the literature, which have been used for the purification of CNTs [45, 69-77]. Other purification methods have also been reported in the literature such as, the electrochemical oxidation of MWCNTs in NaCl or NaBr solution, which leads to halogenation of MWCNTs and the formation of oxygen containing functional groups on MWCNTs [78]; the etching of individual MWCNT in a KCl solution by applying a positive potential higher enough for the occurrence of the electrochemical oxidation [79]; purification by a galvanostatic electrochemical oxidation in H_2_SO_4_. These attributed to the purification mechanism to a faster oxidation rate of amorphous carbon than that of MWCNTs.

The functionalization of CNTs plays a vital role in the preparation of CNT modified electrodes. In one of our work, we followed Yan *et al.* to functionalize MWCNTs with hydrophilic groups [80]. Briefly, the hydrophobic nature of MWCNTs can be converted to a hydrophilic nature by placing MWCNTs (10 mg) and potassium hydroxide (200 mg) in a ruby mortar and grinding them together for 2 hr at room temperature. Then, the reaction mixture is dissolved in double distilled deionized water (10 mL) and then precipitated many times in methanol for the removal of potassium hydroxide. This process not only makes MWCNTs hydrophilic in nature, but this also helps to break down larger bundles of MWCNTs into smaller ones [10]. Numerous other methods have also been reported in the literature for the functionalization of CNTs [81]. After the pretreatment, purification or functionalization procedures, the CNTs must be used on the electrodes for the sensor applications. The electrodes have been modified with CNTs using physical or chemical adsorption or electrochemical deposition. However, because of the CNTs hydrophobic nature there is an important challenge in the preparation of uniformly dispersed CNT solution, which alone should be used to cast on the electrode surface. Different dispersion methods have been reported on the literature, such as dispersion of a sample of purified CNTs (2 mg) in 0.5 wt% Nafion in ethanol (1 mL) for 4 h under ultrasonic agitation [76] or suspension of CNTs (1 mg) in 1 wt % HAuCl_4_ solution (1 mL) by sonication for 5 min to make nanotubes disperse equably, followed by the dilution of the suspended solution to 100 mL with doubly distilled water and heating to boiling while stirring [77]. Similar dispersion methods using ultrasonic agitation and stirring are widely available in the literature [81].

**Figure 1.**
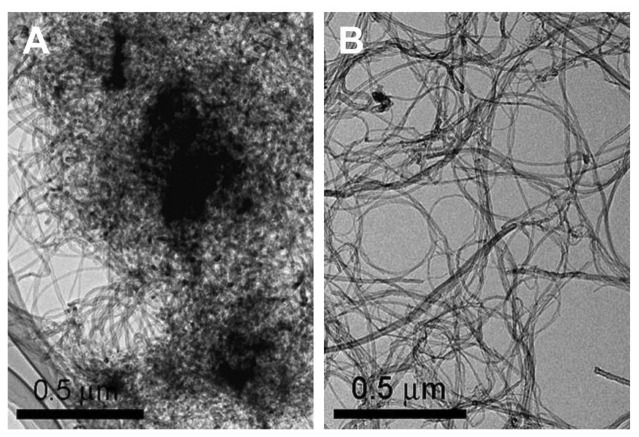
**(a)** Highly entangled as-synthesized MWCNTs; **(b)** Low magnification image of dispersed MWCNTs (5.7 mg dm^-3^). Reproduced from [84] with permission.

Even though numerous methods of dispersion exist, the important factors necessary for a good dispersion are stability of the solution, no damage or modification on the CNT structure, and long term stability of the uniform homogeneous solution. Generally, as reported above, the dispersion of CNTs has been carried out by physical (ultrasonication and milling) and chemical methods (covalent and noncovalent functionalization). These methods cause damage to CNTs and add impurities to it [82, 83]. To overcome these drawbacks, in one of our work, we have followed the previous studies for the dispersion of MWCNTs, which were conducted by K.Y. Chun *et al.* [84]. Briefly, homogeneous dispersion of MWCNTs in ethanol solution was prepared using π-stacking interaction. The chemicals used for making the π-stacking complex were potassium as a doping material, phenanthrene as a nonpolar molecule and 1,2-DME as a dipole solvent. MWCNTs (about 50 mg) were added to a 1,2-DME solution (20 mL) containing 0.2 mol dm^-3^ phenanthrene. The reaction mixture was stirred using a magnetic bar at 400 rpm at room temperature (25° C ± 2) for 48 h. The resulting doped MWCNTs were washed thoroughly with ethanol and water for several times and dried. Then, the obtained MWCNTs (10 mg) in ethanol (10 mL) were sonicated for 30 min to get a uniform dispersion. Figure 1 shows the characterization of the well dispersed CNTs thus prepared.

## 3. Magnetite Nanoparticles with MWCNT and Polypyrrole Hybrid for DNA sensor

Mercaptoacetic acid (RSH) coated magnetite nanoparticles capped with 5′-(NH_2_) oligonucleotide on MWCNTs-COOH/polypyrrole (ppy) modified GCE as an electrochemical biosensor show good results for monitoring DNA hybridization processes [85]. The MWCNTs-COOH/ppy film was electrochemically deposited on the pretreated GCE using 0.1 mol L^-1^ pyrrole, 0.2 mg mL^-1^ MWCNTs and 0.2 mol L^-1^ KCl in PBS (pH 2.0). The potential range used for the electrochemical deposition of polypyrrole was 0 to 0.7 V by CV. The magnetite nanoparticles were prepared by dissolving FeSO_4_·7H_2_O (0.1668 g) and FeCl_3_·6H_2_O (0.27 g) in 200 mL solution with N_2_ purging for 30 minutes. About 1.5 mol L^-1^ ammonia was added slowly into the solution with vigorous stirring until the pH of solution reaches 9.0. The resulting solution would turn to colloidal formation. Then the residue was washed with water and alcohol and suspended in 40 mL alcohol solution. The coating of RSH on the surface of magnetite nanoparticles was done by mixing magnetite nanoparticles solution (5 mL) with RSH (0.4 mL) and keeping it in a nitrogen atmosphere for 8 hours. The resulting solution was washed with alcohol for many times and finally stored in PBS (pH 7.0).

**Figure 2.**
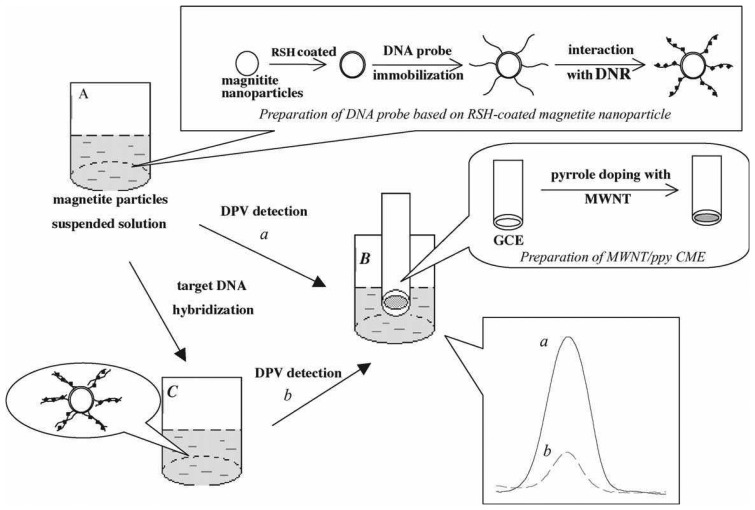
Schematic representation of electrochemical detection of DNA hybridization based on RSH coated magnetite nanoparticles labeled oligonucleotide DNA probe by MWCNTs/ppy GCE. Step A is the preparation of DNA probe, step B is the detection of DNR connected on DNA probe and step C is the hybridization with target sequences and its detection. Reproduced with permission from [85].

For the experiments, human porphobilinogen deaminase (PBGD) promoter 5′-CCT CCA GTG ACT CAG CAC AGG TTC CCC AG-3′ (from 170 to 142) was used as a probe sequence with an amino group at 5′ end. The oligonucleotides were used as target sequences without amino group at 5′ end. The preparation of DNA probe/magnetite nanoparticle conjugate was done by dissolving 5′-NH_2_ capped oligonucleotide in 0.1 mol L^-1^ imidazole solution (100 μL) for 30 min, and then magnetite nanoparticles suspension (5 mL) with 0.008 mol L^-1^ 1-ethyl-3-(dimethylaminopropyl)-carbodiimide hydrochloride (EDAC) was added into the solution. EDAC is a water-soluble coupling reagent used to covalently link oligonucleotides probe (amino group at 5′ end) with the carboxyl groups of MWCNTs. Further, the mixture was stirred for 48 h at room temperature. The unbound oligonucleotides present in the solution were removed using a magnet and the resulting solution was washed with PBS. Then, the magnetite nanoparticle/DNA probe was suspended in PBS (pH 7.4, 5 mL). Then, daunomycin (DNR) solution (100 μL; it is a well known anti-tumor drug by intercalating into GC sequence of DNA) (*c*DNR = 3.15 × 10^-3^ mol L^-1^) was added to DNA probe solution (1 mL) with slow stirring for 15 minutes. The non-bound DNR was eliminated by washing with PBS. Finally, the reduction peak current for DNR on DNA probe was measured by differential pulse voltammetry (DPV). Similarly, the hybridization of target oligonucleotide was added with gentle stirring at 38 °C for 30 min and washed with PBS to remove the partially absorbed ssDNA. Then the DNR reduction peak was measured using DPV. Figure 2 shows the film fabrication process.

The characterization of RSH coated magnetite nanoparticles was done by transmission electron microscopy (TEM) and it shows that most of the particles are quasi-spherical in the average diameter of about 10 nm. The characterization of chemical bonds between the Fe_3_O_4_, RSH and DNA were done by IR spectrometry. The IR results confirmed that the presence of RSH and the hybridization of DNA onto the magnetite particles. The electrochemical behavior of DNR at MWCNTs-COOH/ppy GCE was examined by DPV. The result showed that the reduction peak current for DNR at MWCNTs-COOH/ppy GCE was obvious and the peak potential shifted to negative side. Authors claim that, this result shows the acceleration of electron transport speed and increased active sites on the modified electrode. The optimization of DNA hybridization was examined from complementary sequence oligonucleotide and three-base mismatched sequence of oligonucleotides. In these experiments, the two important factors, temperature and ionic strength influence the oligonucleotide hybridization process. The temperature raise from 28 to 38 °C gradually increased the response for the difference between complementary and three-mismatched sequence and gradually decreased when the temperature rose higher than 40 °C.

The DNA hybridization detection by DNA electrochemical probe is purely based on the DNA duplex match. Similarly, the selectivity is based on RSH-coated magnetite nanoparticles, where the presence of nanometer sized magnetite (10 nm) enhances the selectivity. The selectivity and DNA hybridization detection process showed that the response for non-complementary sequence is the same with DNA probes. Further, this complementary sequence reduced the DNR reduction peak current, which indicates that there is difference between these sequences. The increasing target concentrations of complementary sequences exhibited linear with the concentration of target oligonucleotides. Also, this type of sensor showed very low detection limit comparing with the literature reports. Thus, the DNA probe combined with MWCNTs-COOH/ppy modified electrode has been successfully applied for the detection and determination of the complementary sequence, non-complementary sequence and three bases mismatched sequences.

## 4. Platinum Nanoparticles Combined MWCNTs for DNA Biosensors

**Figure 3.**
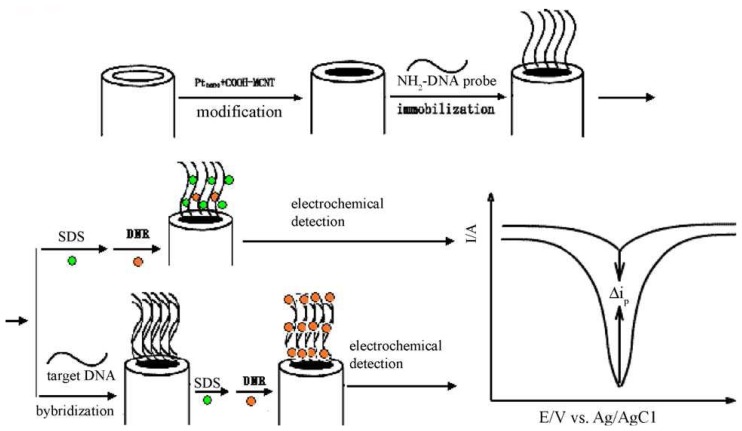
Schematic representation of the electrochemical detection of DNA hybridization based on platinum nanoparticles combined MWCNTs. Reproduced with permission from [86].

Platinum (Pt) nanoparticles were used in combination with MWCNTs for fabricating sensitivity enhanced electrochemical DNA biosensor [86]. The platinum nanoparticles were prepared using sodium citrate method, whereby H_2_PtCl_6_·6H_2_O (4 mL, 5% aqueous solution) was mixed with distilled water and heated at 80 °C with constant stirring, then sodium citrate (60 mL, 1% aqueous solution) was added. The resulting solution was maintained at 80 ± 0.5 °C for about 4 h. This above mentioned reduction process was observed by absorption spectroscopy. The stock solution was prepared by mixing MWCNTs (2.0 mg) with Nafion (100 μL) and Pt nanoparticles solution (900 μL) and sonicating for about 40 min, which forms uniformly dispersed MWCNTs with Pt nanoparticles. Before modification, the electrode was cleaned by cycling potential between -0.5 and +1.2V (versus Ag/AgCl) in 50 mM PBS pH 7.2, until a stable CV profile was obtained. After this process, the modification of GCE was done by dropping MWCNTs/Pt nanoparticles (5 μL) and drying in air. Further, the immobilization of oligonucleotides probe on the MWCNTs/Pt nanoparticles was done by immersing the modified GCE in 2.25 × 10^-5^ mol L^-1^ oligonucleotide solution containing 0.1 mol L^-1^ EDAC and 10 mM acetate buffer (pH 5.2) for 10 h with stirring at room temperature. The electrode was washed well with sodium dodecyl sulfate (SDS) containing PBS to remove the unadsorbed binding. Using this above method, oligonucleotide probes were immobilized on the modified GCE with the formation of amide bonds between -COOH on MWCNTs. The hybridization of DNA on probe captured ssDNA modified MWCNTs/Pt nanoparticles/GCE was obtained by dipping the modified electrode in PBS containing different concentration of target DNA for 30 min at 37 °C. Again, the electrode was rinsed with SDS containing PBS to remove the non-hybridized target DNA. This modified electrode was dipped in PBS containing daunomycin (1.0 × 10^-5^ mol l^-1^) and then washed with PBS to remove the non-adsorbed molecules. Figure 3 shows the detailed electrode modification process.

The presence of MWCNTs with Pt nanoparticles was confirmed by analyzing the film by TEM. The electrochemical characteristics of three different types of films were characterized using CV. The three different films are Pt nanoparticles/GCE, MWCNTs/GCE and MWCNTs/Pt nanoparticles/GCE. From the CV response, authors found that, MWCNTs/Pt nanoparticles have much larger surface area, where larger quantities of ssDNA can be immobilized. This also results in lowering the detection limits of sequence-specific DNA. To find the optimized film condition and hybridization, the Pt nanoparticle solutions were also used in combination with nafion for the solubilization of MWCNTs. From the above experiments, the optimum concentration found for the mixture was 2.0 g L^-1^. Similarly, for the electrode modification 5.0 μL of MWCNTs/Pt nanoparticles was found to be the optimum amount. To prevent the non specific binding, before the intercalator binding process, the ssDNA modified MWCNTs/Pt nanoparticles/GCE was pretreated with 0.2% SDS. Further, the pretreatment of the modified electrode with SDS showed decrease in the redox current of daunomycin. Using this pretreatment, authors prevented the strong non specific interaction between daunomycin and ssDNA along with lowering the detection limit of DNA hybridization.

The electrochemical detection of DNA hybridization was done by DPV technique. The difference in DPV responses between MWCNTs/Pt nanoparticles/GCE (oligonucleotides immobilized film) and MWCNTs/GCE shows that the use of MWCNTs/Pt nanoparticles/GCE possess two times larger electrochemical signals for daunomycin. In selectivity of DNA hybridization, probe/MWCNTs/Pt nanoparticles/GCE showed increase in current value of daunomycin at +0.44 V when hybridized with its complementary sequence. Further, the peak current of DPV for intercalated daunomycin increases with the increasing concentration of complementary target DNA.

These results confirm that MWCNTs/Pt nanoparticles based hybridization showed good selectivity. Furthermore, the results exhibit that MWCNTs/Pt nanoparticles based hybridization assays have high selectivity and wide range of measurements with lower detection limits. The enhancements in the selectivity and detection limits are due to the enhancement in electrical contact between the components in the presence of high surface area Pt nanoparticles. In general, Pt nanoparticles possess high catalytic activities for chemical reactions, so the sensing signal for DNA hybridization is greatly amplified. From these arguments it is confirmed that Pt nanoparticles combined with MWCNTs fabrication has been exhibited as a good electrochemical DNA biosensor.

## 5. MWCNTs and Colloidal Gold Nanoparticles with DNA for the Detection of Effect of Berberine on DNA from Cancer Cells

The chemical modification of screen printed carbon electrode (SPE) with MWCNTs and colloidal gold nanoparticles (GNP) in PBS has been used as the signal transducer of dsDNA based biosensor [87]. This type of biosensor has been used in testing of berberine, with significant antimicrobial and anticancer activity. Berberine has a very strong, concentration dependent, effect on the structural ability of DNA from the human cancer cells (U937 cells). The U937 cells and keratinocyte cells used for the experiments were washed with PBS and lysed with 10 mmol L^−1^ TRIS, 10 mmol L^−1^ EDTA, 0.5 % Triton X-100 mixture solution supplemented with proteinase K (1 mg mL^−1^). After lysis, RNA-ase was added and incubated for 1 hour, which was used for the electrode modification process. The DNA stock solution was manually dropped on the bare SPE surface and the nanostructured films were prepared by layer-by-layer coverage method (DNA/nanomaterial/SPE) and mixed the coverage (DNA-nanomaterial/SPE) and evaporated for overnight. Prior to the first measurement, the DNA modified electrode was immersed in 5 mmol L^−1^ PBS, pH 7.0, for 5 min with constant stirring. After this process, the DNA marker [Co(phen)_3_]^3+^ was deposited by open circuit potential from its 5 × 10^−7^ mol L^−1^ solution in 5 mmol L^−1^ PBS for 120 s with stirring. Further, the cathodic DPV of the marker peak current was measured, evaluated and corrected to blank, and it was reported as negligible. The modified electrode was then regenerated by immersing in high ionic strength PBS solution (0.1 mol L^−1^) for 120 s, with constant stirring to remove the partially accumulated DNA marker ions from the layer. To validate the effect of berberine on DNA, the DNA modified electrode was incubated in berberine solution in PBS with constant stirring and further rinsed with water. The CV of K_3_Fe(CN)_6_ was also recorded and evaluated against blank PBS.

The electrochemical characteristics of DNA marker [Co(phen)_3_]^3+^ was evaluated for different type of SPE modification with the mixture of DNA and nanomaterials. The results showed that higher marker signals were obtained for DNA-GNP/SPE and DNA-(MWCNTs-SDS)/SPE biosensors. The comparison of these results with DNA-(GNP-MWCNTs-SDS)/SPE showed that there is no synergism effect of nanomaterials (biocompatible). The dissociation rate constant evaluation results are indicative that there is some difference between DNA structural arrangement with the marker binding ability on the electrode surface, in the presence or absence of nanostructured materials. This confirms that the stable marker binding with the DNA modified showed great interest for the electron transfer process. The modified electrodes were further examined with K_3_[Fe(CN)_6_] as redox indicator in the solution. The Fe(CN)_6_^3-^ plays as an indicator, and the function based on electrostatic repulsion of Fe(CN)_6_^3-^anion with negatively charged DNA phosphate backbone. From these results authors found that SDS for MWCNTs dispersion is significant despite its negative charge and acts as a barrier for the hexacyanoferrate anion. The bare SPE was characterized by measuring CV in K_3_[Fe(CN)_6_]. This has been compared with DNA modified SPE, which showed worse separation with bare SPE. But the nanomaterials showed better redox reversibility of hexacyanoferrate in presence and absence of DNA. The voltammetric signal for guanine and adenine residues were examined at various types of modified electrodes. GNP-MWCNTs (MWCNTs in DMF or SDS) cause a negative shift of guanine moiety when comparing with DNA/SPE. At the same time, for adenine moiety, the negative shift in oxidation peak occurs for DNA-MWCNTs/SPE, DNA-(MWCNTs-SDS)/SPE and DNA-(GNP-MWCNTs-SDS)/SPE when comparing with DNA/SPE, respectively. This negative shift is because of the electrocatalytic property of MWCNTs.

Before the berberine analysis, authors have examined the stability of human DNA on the modified electrode surface and the results were found satisfactory. The effect of berberine was analyzed by using voltammetric techniques. The concentration of berberine used was in the range of 0.1 to 75 μg mL^-1^. The damage of berberine concentration indicated by the decrease in the DNA marker signal which represents the portion of dsDNA survives incubation in the solution. The interaction of berberine with DNA was the formation of an intercalation complex with dsDNA in low ionic strength solution. From the CV of K_3_[Fe(CN)_6_], it was found that the changes in DNA were found with the increasing concentration of berberine. Also, the changes were greater for DNA from cancer cells than the keratinocytes. Further, at DNA-(MWCNTs-SDS)/SPE the K_3_Fe(CN)_6_ current was measured. The DNA-(MWCNTs-SDS)/SPE sensor incubation affected the DPV signal of the guanine and adenine moieties. For low concentration of berberine (0.1 and 1 μg mL^-1^) there is no effect on the signals from DNA for U937 and kerationcytes. At the same time, the increasing concentration of berberine (75 μg mL^-1^) shows change in guanine and adenine signals, which confirms the greater effect of berberine on DNA from the cancer cells. From the above illustrations authors clearly demonstrated that this type of DNA based biosensor acts as effective chemical toxicity sensor for the rapid detection of DNA damage species, and screening of DNA anticancer agents such as berberine.

## 6. MWCNTs/Nano Zirconium Dioxide/Chitosan for DNA Hybridization Detection

A novel and sensitive electrochemical DNA biosensor based on MWCNTs/nano zirconium dioxide (ZrO_2_)/chitosan (CHIT) modified GCE has been fabricated along with the immobilization of oligonucleotides [88]. Before the film modification process, the zirconia nanoparticles were treated with SDS (100 mL 0.7%) and the pH was adjusted to 4 and stirred for 6 hours. The resulting solution was filtered and washed to obtain the surface treated zirconia nanoparticles. Similarly, the MWCNTs were treated with 3:1 mixture of concentrated H_2_SO_4_ and HNO_3_, which was ultrasonicated for 6 hour to establish the carboxylic acid groups onto the MWCNTs surface. These oxidized MWCNTs were filtered, washed and dried at 100 °C. The CHIT solution was prepared by dissolving the CHIT flakes in 0.05 mol L^−1^ acetic acid with constant stirring for 3 h. The surface treated zirconia nanoparticles and MWCNTs were dispersed in 0.1% CHIT. The mass ratio of the film has been maintained at 1:2.5:100 (ZrO_2_: MWCNTs:CHIT). The resulting mixture was sonicated to obtain a highly dispersed colloidal solution. This colloidal MWCNTs/ZrO_2_/CHIT was casted on the pretreated GCE and dried in air. After drying, the electrode was mounted with probe DNA of 0.5 μL (130 nmol L^−1^) using a pipette. Finally, the modified electrode was washed with PBS to remove the unadsorbed probe DNA. The resulting ssDNA modified electrode was further immersed in hybridization buffer solution for 30 min which contains the target DNA. After this process, the modified electrode was immersed in DNR solution (1.0 μmol L^-1^) and allowed for 15 min for sufficient reaction to occur. After the accumulation process, the modified electrode was washed with PBS and the response signal was measured by CV and DPV in 0.01 mol L^-1^ PBS.

The SEM analysis confirmed the presence of MWCNTs and ZrO_2_. Further, to discern the role of individual components, electrochemical characterization of different type of film has been carried out. For ZrO_2_/CHIT, MWCNTs/CHIT, MWCNTs/ZrO_2_/CHIT the CV response was gradually increased. For ZrO_2_/CHIT film there is no obvious voltammetric peak obtained, which shows that ZrO_2_ has no catalysis for the oxidation of daunomycin (DNR). Also, the introduction of MWCNTs increased the unique catalysis feature and excellent electron transfer ability of the film. The three electrodes (i) ssDNA/ZrO_2_/CHIT/GCE electrode, (ii) ssDNA/MWCNTs/CHIT/GCE electrode and (iii) ssDNA/MWCNTs/ZrO_2_/CHIT/GCE electrode were compared by CV. The comparison shows that the signal obtained for the electrode (a) and (b) were lower than (c). The authors reported that this is because of the synergistic effects of MWCNTs/ZrO_2_/CHIT, which provided the increased loading of ssDNA with good electron transfer ability and high active surface area.

**Figure 4.**
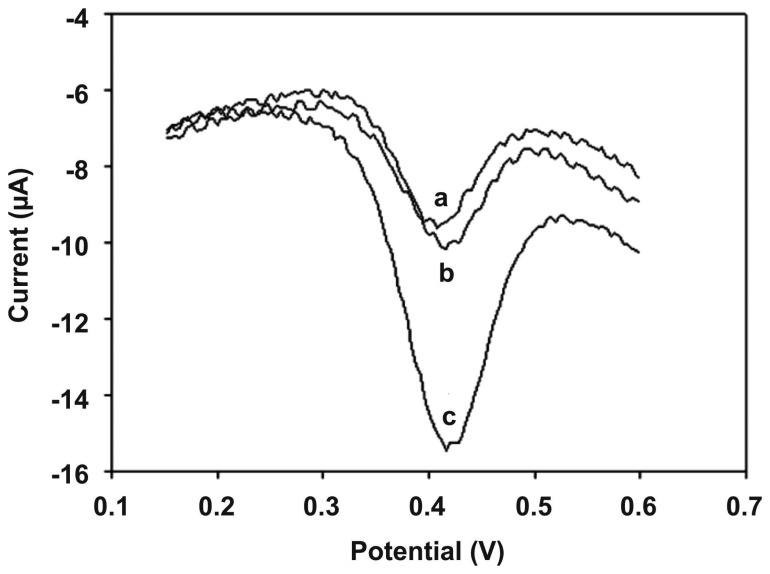
The DPV response of daunomycin as indicator in PBS (pH 7.0) using ssDNA probe modified MWCNTs/ZrO2/CHIT/GCE electrode (a); after exposure to 1.32 × 10^-9^ mol L^-1^ four-base-mismatched DNA sequence (b); the same after hybridization with the complementary target DNA sequence (7.45 × 10^-10^ mol L^-1^) (c). Reproduced with permission from [88].

In this type of biosensor, the hybridization time for obtaining the DPV signals was found as 0.25 to 1.5 h at 60 °C. Based on this, the incubation time was adopted as 30 min in all the experiments. The accumulation time for the increasing peak current of DNR was found as 15 min. From the DPV peak current calibration curves of DNR, it is obvious that the response signal increases linearly with the increase of logarithm of the target DNA concentration. This above mentioned increase was linear with very low detection limit of 7.5×10^-11^ mol L^-1^ (S/N=3). The selectivity of ssDNA/MWCNTs/ZrO_2_/CHIT was found by hybridizing the film with different kinds of DNA sequences. Figure 4 shows the DPV response of ssDNA modified electrode (a), ssDNA modified electrode hybridized with 1.32 × 10^−9^ mol L^−1^ of four base mismatched oligonucleotide (b) and ssDNA modified electrode hybridized with 7.45 × 10^−10^ mol L^−1^ of perfectly matched oligonucleotide (c). The sensor showed small responses during the hybridization with four base mismatched oligonucleotide. Comparing the current ratios, it shows 0.66 for four base mismatch sequence and 1.77 for the complete complementary oligonucleotide concentration. According to the results, the complementary target sequence showed a significant signal increase, which confirms the high selectivity of this type of sensor. Also, the comparison with previous literature reports showed that this DNA sensor is better than the previous reports. Further, the reproducibility was measured for three DNA sensors and it showed that the current response values are within acceptable variable coefficient. This shows the sufficient reproducibility of this type of sensor. These results also show that MWCNTs/ZrO_2_/CHIT composite film shows obvious electrochemical detection.

## 7. Probe Labeled with Silver Nanoparticles Loaded MWCNTs for DNA Hybridization Detection

Gao *et al.* proposed a sensitive electrochemical method for the detection of DNA hybridization based on the MWCNTs with silver nanoparticles (Ag) [89]. The proposed method has been successfully applied for the DNA detection in cystic fibrosis related process. The silver nanoparticles have been prepared in the range of 10-20 nm (average diameter of 15 nm) and stored in dark at 4 °C. The MWCNTs were purified by ultrasonication method in solution containing nitric acid and perchloric acid (7:3), and then activated by purging N_2_ gas for overnight following the addition of 1.5 g of SnCl_2_.2H_2_O with constant stirring. The activated MWCNTs was added to plating solution and then electroless plating reaction was carried out at 25 °C for 15 min with the agitation process. Finally, the suspension of Ag-MWCNTs was filtered and the precipitate was washed with distilled water. In the next step of the process Ag-MWCNTs with DNA sequence as label has been prepared by the self-assembly technique, whereby Ag-MWCNTs (10 mg) was suspended in water (10 mL) and 33 μmol L^-1^ 3′-thiol-modified DNA (0.5 mL) [P1 (3′-HS-ATC CTC AAC TCT-5′) 12 bases or P2 (3′-HS-CTT TTA TAG TAA CCA CAA AG-5′) 20 bases] was added to Ag-MWCNTs suspension solution (1 mL). After standing for 20 h, the PBS concentration of the suspension was gradually adjusted to 0.08 mol L^-1^ by adding aliquots of 1.0 mol L^-1^ PBS. Then, after 6 h the NaCl concentration in the suspension was adjusted to 0.075 mol L^-1^ by adding aliquots of 1 mol L^-1^ NaCl. Further, the concentration of NaCl was readjusted in suspension by adjusting 0.10 mol L^-1^ by adding aliquots of 1 mol L^-1^ NaCl after 2 h and finally allowed to stand for 90 h. Finally, the Ag-MWCNTs-labeled DNA probe was washed with PBS (0.10 mol L^-1^ PBS) and redispersed in PBS (10 mmol L^-1^, 1 mL). The resulting suspension (final concentration of Ag-MWCNTs-labeled DNA probe was 10 g L^-1^) was stored at 4 °C and used as Ag-MWCNTs-labeled DNA probe. The Ag-labeled DNA probe for assessment was prepared in the similar way. The optimization of DNA hybridization has been examined by self-assembled time of target single strand ssDNA. The results exhibited that the response increases with the increase of self-assembled process from 3.5 to 4 h. The influence of concentration of Ag-MWCNTs-labeled DNA probe on the DPV signal was also examined. Similarly, for target sequence, the electrode has been fabricated by immersing the pretreated gold electrode in the target analyte solution for 4 h and the target sequence was immersed in 0.10 mmol L^-1^ 1-hexanethiol (HT) for 1 h to eliminate nonspecific adsorption. Then, the modified electrode was immersed in the probe suspension Ag-MWCNTs-labeled DNA or Ag-labeled DNA for hybridization approximately for 40 min. This hybridized electrode was employed as the working electrode.

The analytical performance of DNA hybridization was investigated and it was found that the peak current increased when using Ag-MWCNTs-labeled DNA probe with the concentration of the target ssDNA. The values are linear with the logarithm of concentration of the complementary target ssDNA. The detection limit was 1 nmol L^-1^ (S/N = 3) for the complementary target ssDNA, which is much lower when compared with that of Ag-labeled DNA probe. The specificity of Ag-MWCNTs-labeled DNA probe for electrochemical detection of DNA hybridization was also examined. The results have shown that there is three bases mismatch with the target DNA in a lower signal. Further, a negligible oxidation signal was obtained for non complementary target DNA. The DPV peak current of target DNA solution is two times higher to that of 3-bases mismatch ssDNA and 20 times greater than that of non complementary ssDNA. From these results, authors found that, the efficient discrimination within the specific binding and non specific binding has been done.

The real application of this type of sensor was for the detection of DNA segments related to the cystic fibrosis. It is one of the most common fatal genetic diseases in European origin and in U.S.A. About 75% cases are responsible by mutation and three missing bases which lead to the deletion of 508^th^ codon. The Ag-MWNTs-labeled DNA resolved the electrochemical detection of mutagenic DNA hybridization. The selectivity of the proposed method is feasible for this type of detection and peak current increased linearly with the complementary target concentration from 3.1 × 10^-14^ to 1.0 × 10^-11^ mol L^-1^. Authors claim that, this range is sufficient enough for the cystic fibrosis samples in clinical diagnosis. The list of various electrodes discussed in this article and the DNA sequences studied at each electrode are given in Table 1.

## 8. Palladium Nanoparticles Combined with MWCNTs for DNA Biosensor

**Figure 5.**
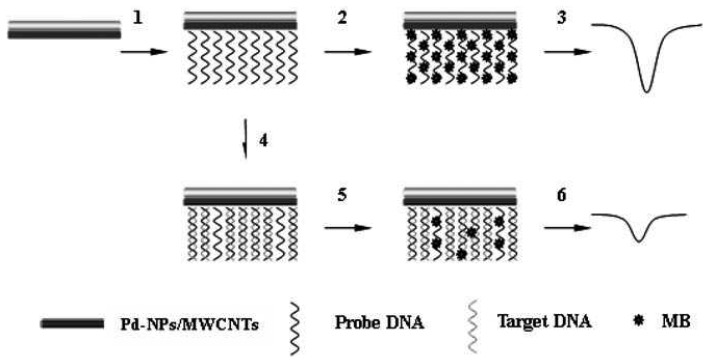
Schematic representation of the electrochemical detection of DNA hybridization based on Pd nanoparticles (Pd-NPs) combined MWCNTs. Reproduced with permission from [90].

Palladium (Pd) nanoparticles combination with MWCNTs, nafion and oligonucleotides (amino groups at the 5′ end) has shown remarkable improvements in electrochemical DNA detection [90]. The palladium nanoparticles were prepared by mixing of Na_2_PdCl_4_ (40.6 mg) in DMF (20 mL) with a solution of NaBH_4_ (75.5 mg) by fast stirring method. The reaction mixture was put under continuous stirring for 24 h, after the mixture reaches deep brown in color. Finally, the precipitate was centrifuged, washed and dried at 60 °C for 24 h. The Pd nanoparticles thus obtained have an average diameter of 3-5 nm. About 8.0 mg of obtained nanoparticles with 1.0 mg MWCNTs were dispersed in the mixture of 100 mL of nafion and 900 mL of DMF, and then sonicated for 60 min. For electrode modification, 5.0 mL prepared solution (MWCNTs/Pd nanoparticles) was immobilized on pretreated GCE and dried in air. The immobilization of oligonucleotide probe was done by the formation of amide bonds between the COOH groups on the MWCNTs and -NH_2_ of the oligonucleotides at 5′. This was carried out by immersing MWCNTs/Pd nanoparticles/GCE in oligonucleotide solution containing 0.1 M EDAC and 10 mM acetate buffer (pH 5.2) for 5 h with continuous stirring. Further, the hybridization process was exhibited by immersing the modified probe DNA of MWCNTs/Pd nanoparticles/GCE into the hybridization solution containing target DNA with constant stirring. Then, the electrode was rinsed to remove unbound DNA and the hybridized electrode was placed in Tris-HCl buffer containing methylene blue (MB) with stirring for 5 minutes. Following the above said process, the electrode was washed with PBS to remove the physically adsorbed MB and the DPV measurements were conducted to measure the electrochemical signal. From the measurement of MB reduction, the target DNA has been detected. Figure 5 explains the electrochemical detection of DNA hybridization based on Pd nanoparticles combined with MWCNTs.

The electrochemical characterization of MWCNTs/Pd nanoparticles/GCE has been carried out in K_3_Fe(CN)_6_ solution and the results showed that MWCNTs/Pd nanoparticles/GCE hold more electronegative surface area than that of MWCNTs/GCE. This has been explained as; Pd-NPs (3-5 nm) hold the capability to adsorb hydrogen produced during the MB redox process, which catalyzes to the oxidation of MB to occur easier than on the Pd-NPs absent modified electrode. The peak current for MWCNTs/GCE exhibits obvious decrease when comparing with MWCNTs/Pd nanoparticles/GCE. Also, MWCNTs/Pd nanoparticles/GCE exhibits larger surface area with probe DNA, which shows the enhanced detection limit for DNA detection. Further, the MWCNTs/Pd nanoparticles/GCE showed higher catalytic activity with MB. In presence of Pd nanoparticles the modified electrode shows the potential shift from -0.375 to -0.321 V. The increasing concentration of Pd nanoparticles increases the peak current of MB, which proves that the reaction of MB was accelerated by Pd nanoparticles. The immobilization of probe DNA was investigated by the signal of MB. After successful immobilization of probe DNA, the peak current of MB was decreased.

This result shows that dsDNA has successfully formed and the interaction of MB with guanine residue was prevented. With three bases mismatch DNA, the decrease in the response of MB reduction is slightly low; this was due to the partially hybridized mismatch DNA bases with the probe. From these results, authors found that the high selectivity of DNA hybridization assay is based on MWCNTs/Pd nanoparticles.

The quantitative detection of DNA hybridization has been carried out from the increasing MB reduction peak current, when increasing the complementary target DNA concentration. Three independent measurements have been carried out, where the average values are linear with logarithmic values of the complementary sequence concentration from 7.5 × 10^-13^ to 2.3 × 10^-9^ M. The detection limit of the complementary sequence was found to be 1.2 × 10^-13^ M (n = 11). The strong interaction between MB and ssDNA exhibits higher electron transfer on MWCNTs/Pd nanoparticles/GCE. From these above results it was found that this new method shows good sensitivity and nonspecific adsorption protocol is obviously dismissed. The summary of sensitivity values and detection limits of various electrodes discussed in this article are given in Table 2.

## 9. Conclusions

In conclusion, the advent of nanotechnology allows man to change the fundamental properties of matter in tailor made materials with desirable attributes, and fabricate functional devices of any dimension. Especially, using simple, however efficient, DNA with CNT and nanoparticle hybrid materials containing sensors has shown their own performance of good stability, fine selectivity and high sensitivity. Also, their electrical and thermal properties are excellent. However, each approach reported here has its advantages and disadvantages. The future development of nanotechnology will embrace various approaches discussed above with their relative contributions depending upon specific applications. Without doubts, these DNA incorporated CNTs and nanoparticles hybrid materials offer, an important step towards the development of selective, down to few target molecules sensitive bio recognition electrodes for various sensor applications. In addition to the above described approaches, there are many other, yet unexplored, strategies in the field of electrochemical and biosensor applications. These strategies are waiting to be explored soon and there is a high expectation among the scientists that such devices would be developed soon, targeting towards the reliable diagnostics of cancer and other diseases.

## Figures and Tables

**Table 1.** DNA sequences studied at different modified electrodes.

Electrode	DNA sequences
Magnetite Nanoparticles-MWCNTs-ppy [85]	Probe: 5′-CCTCCAGTGACTCAGCACAGGTTCCCCAG-3′
	Complementary: 5′-CTGGGGAACCTGTGCTGAGTCACTGGAGG-3′
	Three-base mismatch: 5′-CTGGTGAACCTGTCCTCAGTCACTGGAGG-3′
	Noncomplementary: 5′-AACCCCTTAAACAAAATCAAGTGAATCAA-3′

Pt Nanoparticles-MWCNTs [86]	Probe: 5′-NH2-GAGCGGCGCAACATTTCAGGTCGA-3′
	Complementary: 5′-TCGACCTGAAATGTTGCGCCGCTC-3′
	Noncomplementary: 5′-GAGCGGCGCAACATTTCAGGTCGA-3′

MWCNTs-GNP [87]	Calf thymus dsDNA

MWCNTs-ZrO2-CHIT [88]	Target: 5′-AAAACTTGTGGTAGTTGGAGCTGATGGCGTAGGCAAGAGTGCCC-3′
	Mismatch DNA: 5′-AAATCTTGTGGTAGTTGTAGCTGATGGCGCAGGCAAGAGTGCGC-3′
	Probe DNA: 5′-GGGCACTCTTGCCTACGCCATCAGCTCCAACTACCACAAGTTTT-3′

Ag-MWCNTs [89]	Synthetic (P1): 3′-HS-ATC CTC AAC TCT-5
	Complement of P1: 3′-HS-GTC GTA AGA GTT GAG GAT-5′
	Mismatch of P1: 3′-HS-GTC GTA AGA CTT CAC GAT-5′
	Noncomplementary of P1: 3′-HS-AGC CTT CGG CAT TGC CCC-5′
	Cystic fibrosis: 3′-HS-CTT TTA TAG TAA CCA CAA AG-5′
	Complement of P2: 3′-HS-CTT TGT GGT TAC TAT AAA AG-5′
	Noncomplementary of P2: 3′-HS-AGC CTT CGG CAT TGC CCC-5′

MWCNTs-Pd nanoparticles [90]	Probe: 5′-NH2-GAG CGG CGC AAC ATT TCA GGT CGA-3′
	Complementary: 5′-TCG ACC TGA AAT GTT GCG CCG CTC-3′
	Three-base mismatch: 5′-TCG TCC TGA AAC GTT GCG CCT CTC-3′
	Noncomplementary: 5′-GAG CGG CGC AAC ATT TCA GGT CGA-3′

**Table 2.** Summary of sensitivity values and detection limits of various electrodes.

Electrode	Intercalator	Slope of the linear range (DPV)	Detection limit (pM)	Reference
Magnetite Nanoparticles- MWCNTs-ppy	DNR	0.8255-0.0847c target oligonucotide ×10^13^ (μA)	0.023	[85]

Pt Nanoparticles-MWCNTs	DNR	y = 3.264 log x-3.375 (μA)	10	[86]

MWCNTs-GNP	[Co(phen)^3^]^3+^	Guanine (0.7 and 1.2 μA); adenine (5.4 and 6.1 μA) for U937 cells and keratinocytes respectively	-	[87]

MWCNTs-ZrO2-CHIT	DNR	I = 32.62 + 3.037 log C_DNA_ (M)	75	[88]

Ag-MWCNTs	-	I(0.1 μA) = 4.2 log C - 1.9 (unit of C is 10^-4^ M)	0.010	[89]

MWCNTs-Pd nanoparticles	MB	y = 4.637 log x+9.811 (μA)	0.12	[90]
